# Global research trends in prediabetes over the past decade: Bibliometric and visualized analysis

**DOI:** 10.1097/MD.0000000000036857

**Published:** 2024-01-19

**Authors:** Guoyu Wang, Yafei Chen, Xinxin Liu, Siyi Ma, Min Jiang

**Affiliations:** a Department of Traditional Chinese Medicine, Beijing Shijitan Hospital, Capital Medical University, Beijing, China; b Institute of Basic Research in Clinical Medicine, China Academy of Chinese Medical Sciences, Beijing, China.

**Keywords:** bibliometric analysis, Bibliometrix, CiteSpace, pre-diabetes, research hotspots, visualization, VOS viewer

## Abstract

Object: This study aimed to investigate global research advances and hot trends in prediabetes in the last decade based on a bibliometric analysis of publications. Publications from 2013 to 2022 were retrieved from the Web of Science Core Collection database through a topic search. With the use of CiteSpace, VOS viewer, and Bibliometrix R software packages, the number of publications, production categories, countries/regions, institutions, authors, journals, references, and keywords were comprehensively analyzed to sort out the hot spots and directions of prediabetes and predict the future research directions. A total of 13,223 papers were recruited for this study by the end of March 3, 2023. A generally increasing trend was observed in the number of annual publications. PLOS ONE (journal), USA (national), and the University of Copenhagen (institutional) published the most papers in this research area. The top 3 contributor authors were Tuomilehto Jaakko, Rathmann Wolfgang, and Peters Annette. “Intestinal microbiota” (2020–2022) was the most populated keyword in terms of intensity, and “biomarkers,” “gut microbiota,” and “metabolomics” were the most populated keywords in the last 3 years. “Prediabetes: a high-risk state for diabetes development-2012” was the strongest burst reference. This study summarized the research hotspots and trends in prediabetes research in the last decade. Frontier research can be found in the journal Diabetes Care and Journal of Clinical Endocrinology Metabolism. Prediabetes research focuses on preventing risk factors to reduce the prevalence of prediabetes, and current research hotspots focus on gut microbes and metabolism-related biomarkers.

## 1. Introduction

Prediabetes is the stage of abnormal glucose metabolism between diabetes and normal blood glucose level and mainly includes 2 states, namely, impaired fasting glucose (IFG, fasting plasma glucose of 5.6–6.9 mmol/L) and impaired glucose tolerance (IGT, postload plasma glucose of 7.8 to 11.0 mmol/L based on 2 hours oral glucose tolerance test). The American Diabetes Association (ADA) has a lower threshold for IFG compared with the WHO but the same threshold for IGT. The ADA also includes hemoglobin A1c (HbA1C) levels of 5.7% to 6.4% as a new high-risk category for diabetes. In 2021, about 541 million of adults have IGT and about 319 million have IFG; by 2045, these numbers are projected to increase by 11.4% and 6.9%, respectively.^[1]^ This condition imposes a great burden on the society and economy; thus, early diagnosis and control is necessary. In light of this, the ADA 2022 guidelines pointed to a trend toward younger diabetes, bringing the age of diabetes screening earlier from 45 to 35 years of age, with or without diabetes risk factors such as obesity,^[2]^ and emphasizing the identification of people at risk for prediabetes. Therefore, a comprehensive quantitative analysis of the current situation, focus fields, and prospects for prediabetes is critical for developing effective prevention and control strategies.

Bibliometrics, a discipline that has been around since the early 20th century,^[3]^ has evolved over time and is now an indispensable tool for researchers across various fields. By using mathematical and statistical methods to analyze metadata associated with published journals or data sets, such as titles, abstracts, and keywords, bibliometrics can uncover valuable insights into the trends and connections between publications. Through the analysis of bibliometric data, researchers can gain a deeper understanding of research bases, hot spots, and emerging areas in their respective fields.

Despite the abundance of bibliometric articles on diabetes and its complications,^[4–6]^ the field of prediabetes research has yet to be comprehensively analyzed in this way. Our study fills this gap by using advanced bibliometric tools to systematically evaluate the last decade of prediabetes research. By applying CiteSpace, VOS viewer, and Bibliometrix R package, we are able to conduct a comprehensive analysis of the volume of publications, productive categories, countries/regions, institutions, authors, journals, references, and keywords, thus shedding light on the research hotspots and future directions in this field. This study provides an invaluable reference for clinicians and researchers seeking to advance the understanding and management of prediabetes.

## 2. Methods

### 2.1. Data source and search strategy

The Web of Science Core Collection (WOS) has been recognized by many researchers as a high-quality digital bibliographic resource database covering a large number of publications in many fields and is considered the most suitable database for bibliometric analysis. In this study, the WOS was selected as the data source. For accurate and comprehensive search data, the index was selected as Science Citation Index Expanded (SCI-Expanded), the search formula was TS= (“prediabetic state” OR “prediabetes” OR “prediabetic” OR “pre-diabetes” OR “pre-diabetic stage” OR “pre-diabetes stage” OR “impaired glucose tolerance” OR “impaired fasting glucose”), the time span was 2013 to 2022, the time ended on March 10, 2023, the literature type was “Articles” and “Review Articles,” and the language type was “English.” Then, we removed articles that deviated from the study topic by abstract and title, as and cleaned up the duplicate articles (Table 1).

**Table 1:** TS search quires and refinement procedure.

Set	Results	Refinement
1	30956	TS= (“prediabetic state” OR “prediabetes” OR “prediabetic” OR “pre-diabetes” OR “pre-diabetic stage” OR “pre-diabetes stage” OR “impaired glucose tolerance” OR “impaired fasting glucose”)
2	15763	Refined by Publication Years: (2022 or 2021 or 2020 or 2019 or 2018 or 2017 or 2016 or 2015 or 2014 or 2013)
3	13621	Refined by Document Types: (Article or Review Article)
4	13404	Excluded Document Types: (Early Access or Proceeding Paper or Book Chapters or Retracted Publication)
5	13241	Refined by Languages: (English)
6	13223	Filtered the titles and abstracts of articles to remove those with less relevance

### 2.2. Bibliometric and visualized analysis

EXCEL 2021 was utilized to create a comprehensive graph to capture the growth trend of the literature by year. For an in-depth understanding of the field and its development, 3 powerful applications were employed: CiteSpace (version 6.1), VOS viewer (version 1.6.18), and R language. Each application provided unique advantages, and together they offered a comprehensive analysis of the field.

CiteSpace uses a data normalization method based on set theory for similarity measurement of knowledge units. The algorithm provides Timezone and Timeline views within time slices, clearly outlining the evolution of knowledge and the historical span of literature in a certain cluster. It offers a valuable tool to understand the development process and trends in the field.^[7]^

VOS viewer uses a probability theory method for data normalization and offers various visualization views in the fields of keywords, co-institutions, and coauthors. Its network visualization, overlay visualization, and density visualization features make it simple to create attractive and informative figures.^[8]^

Bibliometrix, an R package, offers all primary bibliometric analysis methods for quantitative research in scientometrics and bibliometrics. Developed in the statistical computing and graphic R language, Bibliometrix follows a logical bibliometric workflow, providing an organized and efficient approach to data analysis.^[9]^

## 3. Results

### 3.1. Overview of prediabetes publications

**Figure 1.**
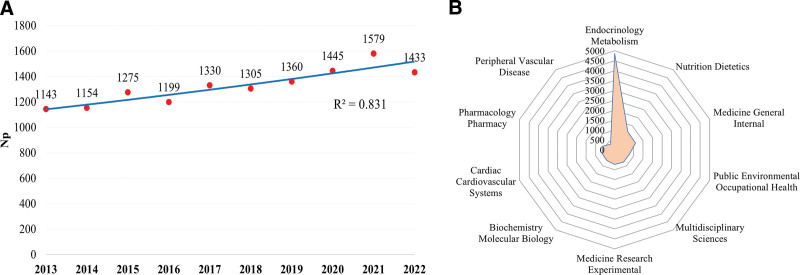
(A) The annual trends in the number of publications (red dots) and curve fitting of growth trends (blue line). (B) Radar map of the top 10 research productive categories on pre-diabetes.

The 13,223 papers used in this study were written by 69,239 authors from 13,350 organizations in 159 countries, published in 1964 journals, and cited 330,085 references from 29,489 journals. According to the polynomial fitting curve, the annual publications of articles on prediabetes fluctuated in the past decade, but the general trend was a gradual increase (Figure 1A, a correlation coefficient R^2^ = 0.831). In particular, the number of articles published after 2020 increased rapidly. Up to 1576 articles were published in 2021, and the number of articles published annually after 2017 was stable at more than 1300. This finding indicated that prediabetes research has attracted the attention of scholars and become a research hotspot in the field.

Figure 1B shows the top 10 research productive categories on prediabetes. The most frequent study category of prediabetes was Endocrinology Metabolism (4873 papers), accounting for about one-third of the total publications, followed by Nutrition Dietetics (1126 papers) and Medicine General Internal (1098 papers).

### 3.2. Bibliometric analysis of countries

**Figure 2.**
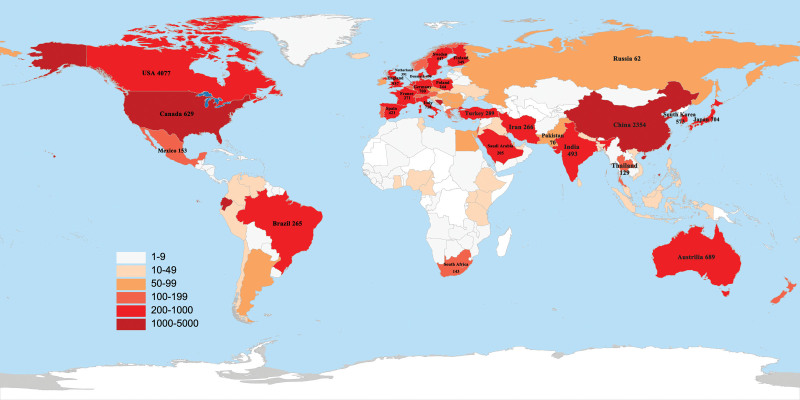
Geographic distribution of pre-diabetes research worldwide.

**Figure 3.**
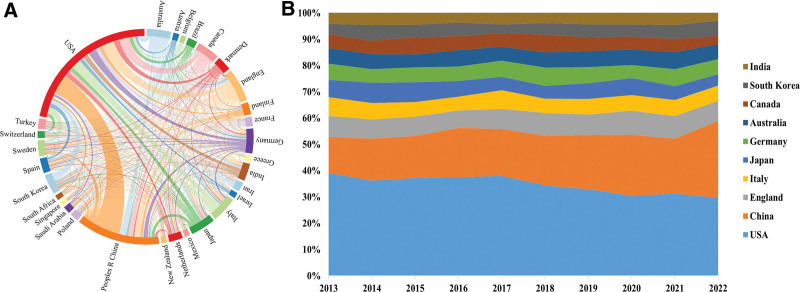
(A) The collaboration network map of countries/regions. (B) The publication trend chart of the top 10 countries in the past decade.

The geographic distribution of the total number of papers on prediabetes research in all countries and regions (Fig. 2). The top 2 countries accounted for almost half of the 13,223 articles published. Research on prediabetes was uneven across countries. Table 2 shows the top 10 countries in terms of the number of published articles for further analysis of the most productive countries in this field. The United States published the highest number of papers (4077, 30.84%), followed by China (2354, 17.8%), England (937, 7.09%), Italy (718, 5.43%), and Japan (704, 5.32%). In addition, the United States had the highest H index (139), followed by England (91) and China (79). Germany also had a high H index (68), which may be related to the high number of German authors ranked in the top 10 of the article production. With regard to the collaboration of the top 30 countries with the highest article quantity (Fig. 3A), the USA was the central core of the collaboration, with the USA, China, and the England collaborating closely in the prediabetes period. The stacked figure (Fig. 3B) showed a decreasing trend in the annual number of articles published in the United States. By contrast, China showed an increasing trend. The annual number of articles published in other countries had grown slowly in the last decade. Therefore, the United States and China were the major drivers for the development of prediabetes research.

**Table 2:** The top 10 countries/regions with the highest productivity.

Rank	Country/Region	Np	Percentage (%)	Tc	H-index
1	USA	4077	30.83	129206	139
2	CHINA	2354	17.80	45207	79
3	ENGLAND	937	7.09	41116	91
4	ITALY	718	5.43	25435	68
5	JAPAN	704	5.32	13833	52
6	GERMANY	700	5.29	24808	68
7	AUSTRALIA	689	5.21	34840	69
8	CANADA	629	4.76	22165	62
9	SOUTH KOREA	573	4.33	15840	42
10	INDIA	493	3.73	13563	51

NP = number of publication, TC = total citation.

### 3.3. Bibliometric analysis of institutions

Table 3 shows the top 10 highly publication institutions. The institution with the highest publications was the University of Copenhagen (161 papers), followed by Shanghai Jiao Tong University (126 papers) and Harvard Medical School (125 papers). University of Copenhagen had the highest Np, Tc and H index, indicating that it published a substantial number of basic research on prediabetes. Four of the top 10 institutions with highest number of publications belonged to the USA, and Harvard Medical School and Johns Hopkins University had a high H index. This finding explained why the USA had the highest number of publications and H index.

**Table 3:** The top 10 highly publication institutions.

Rank	Affiliation	Country	Np	Tc	H-index
1	University Of Copenhagen	Denmark	241	10900	51
2	Harvard Medical School	USA	220	16705	68
3	Shanghai Jiao Tong University	China	204	4772	26
4	Johns Hopkins University	USA	175	7215	35
5	University Of Toronto	Canada	170	4133	32
6	Karolinska Institutet	Sweden	170	4286	30
7	University Of Michigan	USA	169	3486	30
8	Univ Helsinki	Finland	167	5950	32
9	Univ Colorado	USA	152	7247	43
10	Univ Washington	USA	151	7669	45

**Figure 4.**
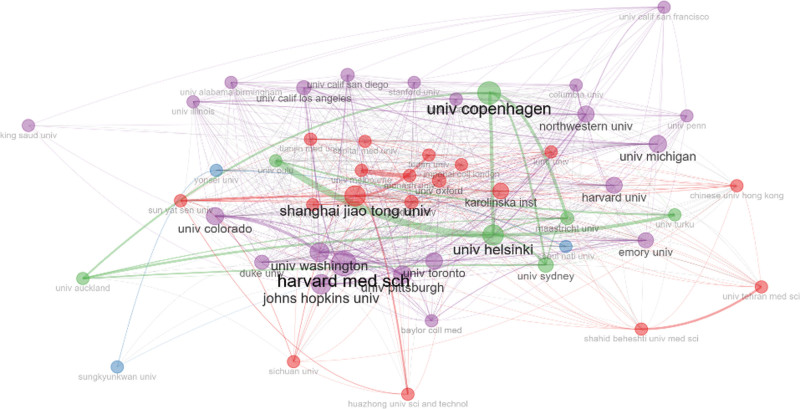
Cooperation network of top 50 institutions.

The visualized network of co-authorship institutions was analyzed via Bibliometrix (Fig. 4). The nodes represent institutions (the larger circle, the higher the number of papers published), and the line represents the collaboration of the institutions (the wider line, the more frequency of collaborations). Great cooperation was observed between institutions. Harvard Medical School preferred to collaborate with multiple institutions, and the University of Copenhagen worked closely with the University of Helsinki, Maastricht University, and The University of Sydney.

### 3.4. Bibliometric analysis of journals

**Figure 5.**
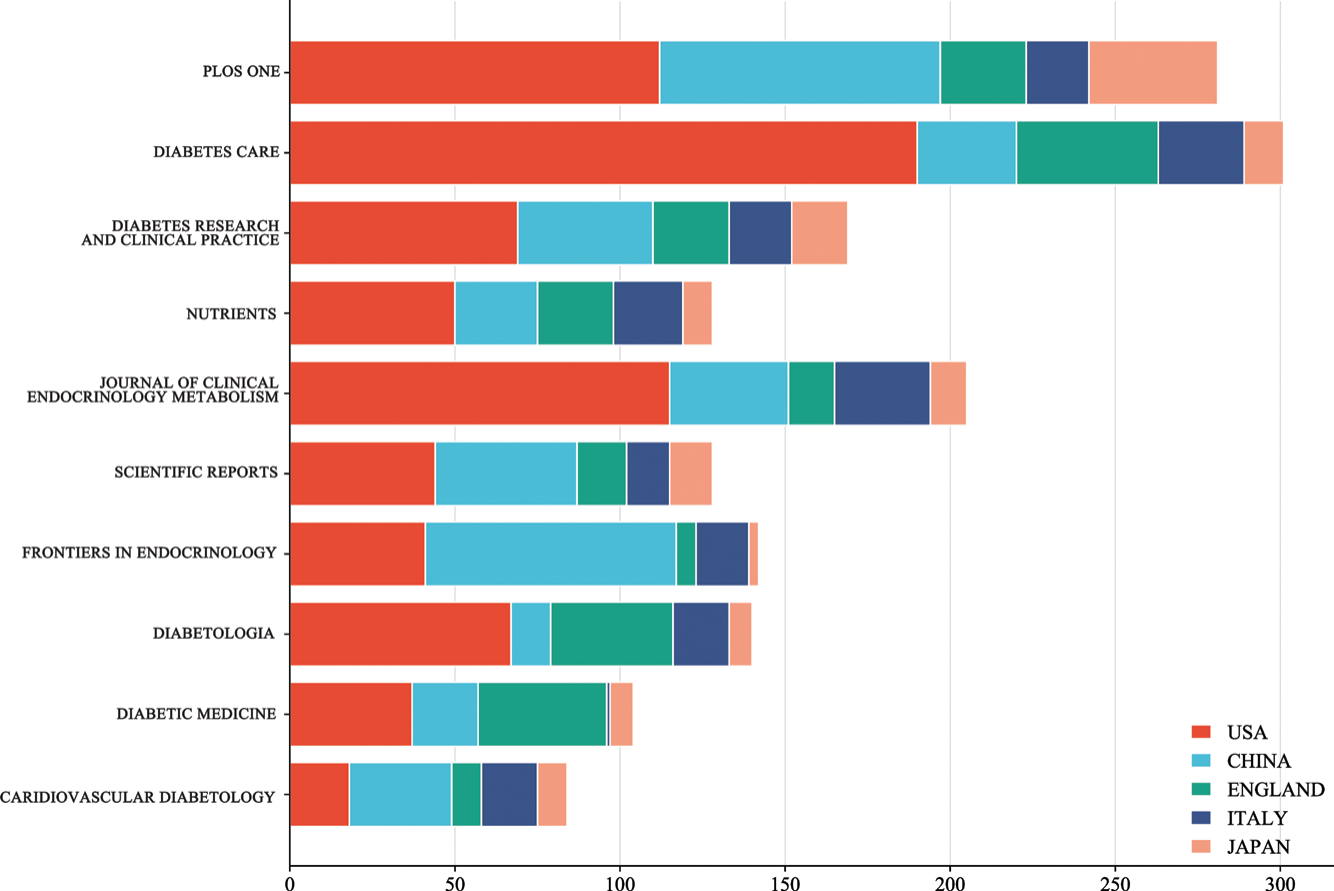
The number of publications by countries in the journals.

Table 4 shows the top 10 journals publishing prediabetes research. Approximately 18% (2377) of the publications were in the top 10 journals. *PLOS ONE* published the highest number of articles researched on prediabetes (448 papers, IF:3.571), followed by *DIABETES CARE* (323 papers, IF:17.155), and *DIABETES RESEARCH AND CLINICAL PRACTICE* (272 papers, IF: 8.18). *DIABETES CARE* had the highest number of total citations and high IF and H indexes, indicating that it published the highest quality articles and received substantial attention for research in prediabetes. Although the publication quantity of *JOURNAL OF CLINICAL ENDOCRINOLOGY METABOLISM* and *DIABETOLOGIA* was not great, they had a high H index and the study was valuable. Figure 5 shows the number of publications by countries in the journals. USA had the most articles in *DIABETES CARE*, demonstrating that this country values this journal and has excellent scientific research base and strength. These results provided some reference and guidance to prediabetes researchers on which journals to publish their findings in the future.

**Table 4:** The top 10 journals in number of articles published.

Rank	Country/Region	Np	Tc	H-index	IF (2021)
1	PLOS ONE	448	10041	48	3.752
2	DIABETES CARE	323	19631	68	17.155
3	DIABETES RESEARCH AND CLINICAL PRACTICE	272	15751	35	8.18
4	NUTRIENTS	238	3026	24	6.706
5	JOURNAL OF CLINICAL ENDOCRINOLOGY METABOLISM	237	7590	45	6.134
6	SCIENTIFIC REPORTS	211	3548	30	4.997
7	FRONTIERS IN ENDOCRINOLOGY	198	1762	22	6.055
8	DIABETOLOGIA	176	5744	40	10.46
9	DIABETIC MEDICINE	147	2860	27	4.213
10	CARIDIOVASCULAR DIABETOLOGY	127	2458	27	8.949

### 3.5. Bibliometric analysis of authors

Analysis of the authors of the literature reveals the representative scholars and core research strengths of the research area. In 1963, the famous scholar price proposed Lotka theorem, that is, half of the papers on the same topic are written by a group of highly productive authors, and this set of authors is numerically equal to the square root of the total number of all authors.


$$\begin{array}{l} \underset{m+1}{\overset{I}{\sum }}\;n\left(x\right)=\sqrt{N} \end{array}$$

where *n(x*) denotes the number of authors who have authored *×* papers, and *I = n*_*max*_ is the number of papers by the most prolific authors in the field. *N* is the total number of authors, and *m* is the minimum number of publications by the core authors. According to Price Law, the minimum number of core authors in a field is *m* = 0.749 × $\sqrt{n_{max}}\approx 6.08$. Therefore, authors with more than 7 publications (including 7) are classified as core authors in the field.

**Figure 6.**
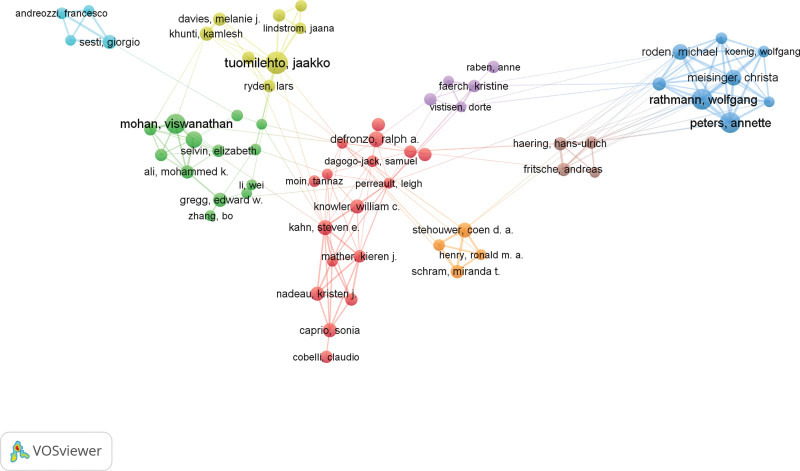
The cooperation network of top 50 authors.

A total of 1154 core authors published 12,095 articles, accounting for 91.47% of the total number of articles. This number reached the 50% standard proposed by Price, and the calculation by substituting the above values into the formula was in line with the formula of Price Law. Therefore, a stable collaborative group of authors has been formed in the field of prediabetes research. Table 5 shows the top 12 highly productive authors in the field in terms of number of publications. Tuomilehto, Jaakko from Karolinska Institute was the most prolific author in this field, publishing 65 articles. His research had received substantial attention. The next authors were Rathmann Wolfgang, Peters Annette, Mohan Viswanathan, and Azizi Fereidoun who published 59, 59, 49, 41, and 39 articles, respectively. Moreover, the top 12 authors were mainly from Germany, China, and USA, indicating that these 3 countries have many outstanding researchers in the prediabetes period. The cooperation network of authors who published articles more than 20 showed that the researchers involved in prediabetes collaborated with each other in the 8 main clusters of collaboration (Fig. 6). The clustering showed a significantly strong domestic collaboration, with cross-country collaboration evident among the impactful researchers.

**Table 5:** The top 15 highly productive authors.

Rank	Author	Affiliation	Country	Np	Tc	H-index
1	Tuomilehto, Jaakko	Karolinska Institute	Sweden	65	4172	26
2	Rathmann, Wolfgang	German Diabetes Center	Germany	59	6080	23
3	Peters, Annette	German Research Center for Environmental Health	Germany	59	3012	22
4	Mohan, Viswanathan	Madras Diabetes Research Foundation	India	49	4502	22
5	Azizi Fereidoun	Shahid Beheshti Univ Med Sci	Iran	41	2374	17
6	Ning, Guang	Shanghai Jiao Tong University	China	39	5437	18
7	Wang Weiqing	Shanghai Jiao Tong University	China	34	2865	13
8	Roden Michael	German Diabetes Center	Germany	34	1250	21
9	Stehouwer Coen D. A.	Maastricht University	Netherlands	32	1002	16
10	Meisinger Christa	German Research Center for Environmental Health	Germany	28	888	18
11	Gregg, Edward W	Centers for Disease Control and Prevention	USA	28	5152	21
12	Caprio, Sonia	Yale University	USA	28	1075	17

### 3.6. Analysis of highly cited articles

The top 10 highly cited articles listed in Table 6 were published from 2013 to 2016. Seven articles had been cited more than 100 times and published in JAMA(IF:157.375), Lancet (IF:202.731), Eur Heart J. (35.855), Diabetes Care (IF:17.155), and Diabetologia (IF:10.46). According to a meta-analysis of the range of HbA1c-defined prediabetes, the article (title: Progression rates from HbA1c 6.0% to 6.4% and other prediabetes definitions to type 2 diabetes: a meta-analysis, LC:112) concluded that progression rates vary by prediabetes definition, which has implications for the planning and implementation of diabetes prevention programs. Meanwhile, HbA1c 6.0% to 6.4% may identify individuals at lower risk of diabetes than other prediabetes definitions.^[10]^

**Table 6:** The top 10 highly cited articles.

Rank	Document	Year	LC	IF (2021)
1	Xu Y, Wang L, He J, et al Prevalence and control of diabetes in Chinese adults. JAMA. 2013;310(9):948–959. doi:10.1001/jama.2013.168118	2013	301	157.375
2	Menke A, Casagrande S, Geiss L, et al Prevalence of and Trends in Diabetes Among Adults in the United States, 1988-2012. JAMA. 2015;314(10):1021-1029. doi:10.1001/jama.2015.10029	2015	201	157.375
3	Zhou B, Lu Y, Hajifathalian K, et al Worldwide trends in diabetes since 1980: a pooled analysis of 751 population-based studies with 4.4 million participants. Lancet. 2016;387(10027):1513–30. Doi: 10.1016/S0140-6736(16)00618-8	2016	163	202.731
4	Rydén L, Grant PJ, et al ESC Guidelines on diabetes, pre-diabetes, and cardiovascular diseases developed in collaboration with the EASD. Eur Heart J. 2013;34(39):3035-3087. doi:10.1093/eurheartj/eht108	2013	131	35.855
5	Dunkley AJ, Bodicoat DH, Greaves CJ, et al Diabetes Prevention in the Real World: Effectiveness of Pragmatic Lifestyle Interventions for the Prevention of Type 2 Diabetes and of the Impact of Adherence to Guideline Recommendations A Systematic Review and Meta-analysis. Diabetes Care. 2014;37(4):922-33. doi:10.2337/dc13-2195	2014	121	17.155
6	Lindstrom J, Peltonen M, Eriksson JG, et al Improved lifestyle and decreased diabetes risk over 13 years: long-term follow-up of the randomized Finnish Diabetes Prevention Study (DPS). Diabetologia. 2013;56(2):284-93. doi:10.1007/s00125-012-2752-5	2013	116	10.46
7	Morris DH, Khunti K, Achana F, et al Progression rates from HbA(1c) 6.0–6.4% and other prediabetes definitions to type 2 diabetes: a meta-analysis. Diabetologia. 2013;56(7):1489–93. doi: 10.1007/s00125-013-2902-4	2013	112	10.46
8	Kautzky-Willer A, Harreiter J, Pacini G. Sex and Gender Differences in Risk, Pathophysiology and Complications of Type 2 Diabetes Mellitus. Endocrine Reviews. 2016;37(3):278–316. doi:10.1210/er.2015-1137	2016	88	25.261
9	Schellenberg ES, Dryden DM, Vandermeer B, et al Lifestyle Interventions for Patients with and at Risk for Type 2 Diabetes A Systematic Review and Meta-analysis. Annals of Internal Medicine. 2013;159(8):543-+. doi:10.7326/0003-4819-159-8-201310150-00007	2013	72	51.598
10	Anjana RM, Rani CSS, Deepa M, et al Incidence of Diabetes and Prediabetes and Predictors of Progression Among Asian Indians: 10-Year Follow-up of the Chennai Urban Rural Epidemiology Study (CURES). Diabetes Care. 2015;38(8):1441-8. doi:10.2337/dc14-2814	2015	71	17.155

LC = local citations.

**Figure 7.**
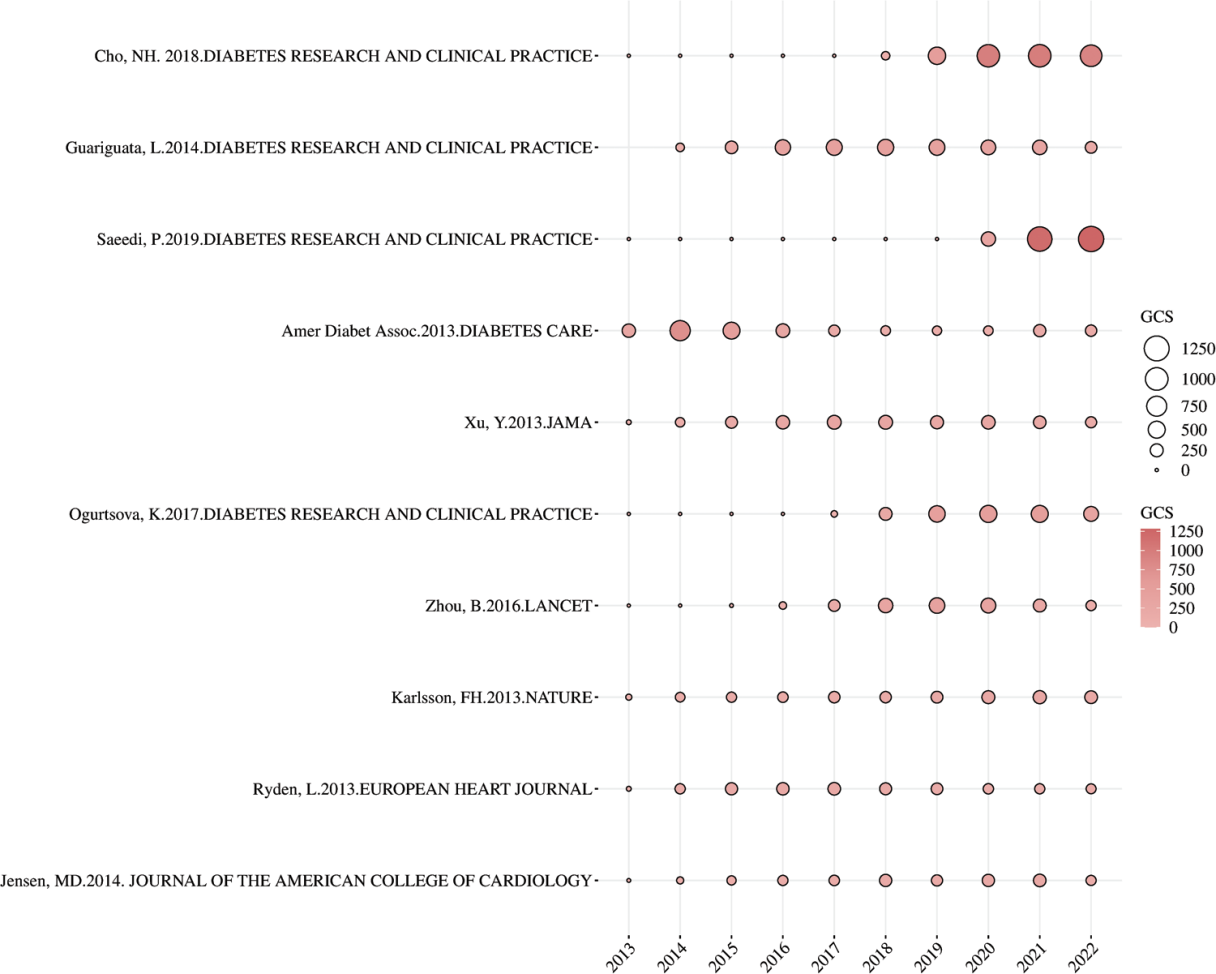
The yearly number of global citations for papers having a high global citation score (GCS).

Figure 7 depicts the annual global citation counts of papers with global citation scores (GCSs). The article with the highest GCS was from Cho NH (GCS = 3444), whose study was mainly based on the 2017 International Diabetes Federation data on global diabetes projections for 2045. The projection is about 693 million cases of diabetes worldwide in 2045, with an estimated failure to diagnose diabetes in time for about half of the patients. Women in pregnancy will be affected by hyperglycemia.^[11]^ Saeedi article had high GCS in 2021 (1190) and 2022 (1283). This study focused on global and regional diabetes prevalence estimates and projections for 2030 and 2045 and reported nearly 500 million people worldwide with diabetes and 374 million people with impaired glucose tolerance in 2019. These values are projected to increase by 25%. The high GCS articles are essentially projections of the future global prevalence of diabetes and prediabetes, indicating that researchers are concerned about the prevalence of diabetes and the urgency of current interventions for prediabetes.^[12]^ The authors of 5 of the articles with the highest global citation scores were from Australia, which may explain the high H index in this country.

### 3.7. Analysis of co-cited references

**Figure 8.**
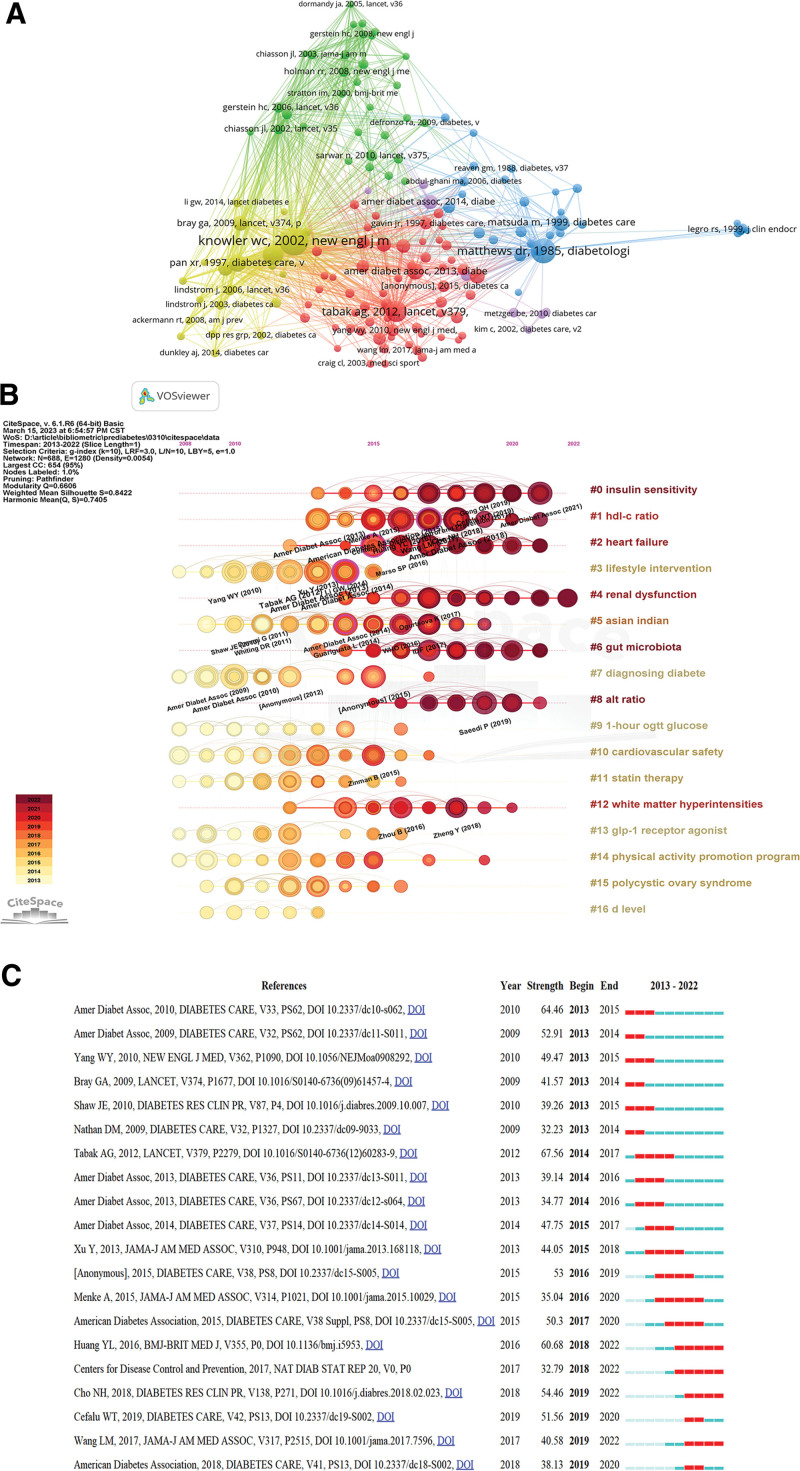
Mapping based on co-cited references from prediabetes research. (A) The network of co-cited references. Of 330,085 references, 149 were cited at least of 100 times (clustered in 5 clusters). (B) The timeline distribution of 11 clusters. (C) Top 20 references with the strongest citation bursts.

The co-cited references emphasize closely related research topics in specific fields. We performed a cluster analysis of the references with over 100 co-citations and found that they can be divided into 5 categories (Fig. 8A). Cluster 1 (red) contains 59 articles mainly concerned with the risk of prediabetes. Cluster 2 (green) is mainly about the prevention of diabetes and prediabetes with 31 articles. Cluster 3 (blue) has 30 references and focuses on model assessment and beta cell function in diabetes and prediabetes. Cluster 4 (yellow) is about the diagnostic and therapeutic aspects of diabetes and prediabetes with 21 reference references. Cluster 5 (purple) comprises 8 references and focuses on gestational diabetes and T2DM.

The Modularity (Q) value in Figure 8B was 0.6606, and the Weighted Mean Silhouette (S) was 0.8422. Q > 0.3 means that the clustering structure is significant, S > 0.5 clustering class is reasonable, and S > 0.7 means that the clustering is convincing.^[13]^ The co-cited references were divided into 17 clusters: #0 insulin sensitivity, #2 heart failure, #3 lifestyle intervention, #4 renal dysfunction, #5 Asian Indian, #6 gut microbiota, #7 diagnosing diabetes, #8 alt ratio, #9 1-hour ogtt glucose, #10 cardiovascular safety, #11 statin therapy, #12 white matter hyperintensities, #13 glp-1 receptor agonist, #14 physical activity promotion program, #15 polycystic ovary syndrome, and #16 d level. Figure 8C illustrates the top 20 references with the strongest citation burst. The reference citation started in 2013, with 4 articles consistently cited through 2022 and 3 articles with 5 years of continuous citations. The strongest citation burst reference was published on *Diabetes Care* by ADA in 2018 (strength: 71.48). Tabak articles also had strong citation burst (strength: 67.56) and was published on *Lancet* in 2012. The most bursting reference in the last 5 years was the article by Huang that focused on a systematic evaluation and meta-analysis of the relationship between prediabetes and cardiovascular disease risk and all-cause mortality. They found that people with fasting blood glucose concentrations as low as 5.6 mmol/L or HbA1c of 39 mmol/mol may have an increased risk of cardiovascular disease.^[14]^ Wang article is also being referenced to date and predicted a 10.9% prevalence of diabetes and a 35.7% prevalence of prediabetes among Chinese adults in 2013 from a cross-sectional survey with ethnic differences.^[15]^

### 3.8. Analysis of author key words

**Figure 9.**
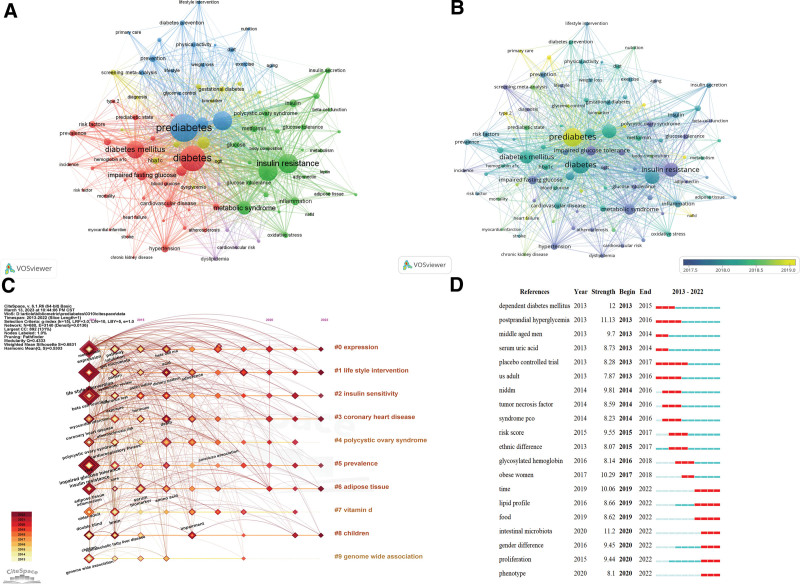
The key words of pre-diabetes mapping. (A) The network of keywords, the 94 terms that appared more than 50 times. (B) Keyword visualization according to the APY. The different colors indicate the relevant year of publication. Yellow keywords came later than purple keywords. (C) The timeline distribution of 10 clusters. (D) Top 20 key words with the strongest citation bursts.

The data were imported into VOS viewer for keyword co-occurrence analysis, and 5 clusters were generated by software clustering (Fig. 9A). Cluster 1 (red) focuses on the risk, complication, and prevalence of diabetes. Cluster 2 (green) is mainly about the pathogenesis and risk factor of prediabetes. Cluster 3 (blue) includes the lifestyle invention and primary care about prediabetes. Cluster 4 is chiefly about IGT and metabolic syndrome with pregnancy. Cluster 5 (purple) has relatively few keywords. All the keywords were color categorized by VOS viewer according to their average year of publication (APY). The most recent keyword was “biomarkers” (cluster 4, 2019.96), followed by “gut microbiota” (cluster 2, 2019.66) and “metabolomics” (cluster 4, APY:2019.62), which are popular topics in the field. In addition, “expression,” “lifestyle interventions,” “insulin sensitivity,” “prevalence,” and “children” have continuously been the research focus of prediabetes (Q = 0.4333, S = 0.683, Figure 9C). Figure 9D illustrates the top 20 key words with the strongest citation bursts. The terms “intestinal microbiota,” “lipid profile,” “gender difference,” “proliferation,” and “phenotype” were trending keywords in the last 3 years.

Figures 9A to 9D show that recent research hotspots focus on intestinal microbiota and biomarkers. Academics will further explore the prevention and treatment of prediabetes from the perspective of metabolism in the future.

## 4. Discussion

Prediabetes, a hyperglycemic state with insulin resistance and impaired beta-cell function, is an intermediate stage between normoglycemia and diabetes. The prevalence of this disease is increasing year by year, and intervention for prediabetic patients to delay or prevent the onset of diabetes is clinically meaningful. Therefore, we systematically reviewed the research status and trends in the field based on CiteSpace, VOS viewer, and R language among the relevant studies about the field of prediabetes published in the WOS in the last decade. Discussion and analysis of the core authors, high productivity countries and institutions, critical journals, and key clusters in the field of research were also conducted.

### 4.1. Research trends in prediabetes

Through polynomial curve analysis, we found that the number of publications on prediabetes research in the last decade had minor fluctuations but showed an overall upward trend. This finding indicated that prediabetes has attracted increasing attention from researchers.

According to its global geographic distribution, prediabetes research existed worldwide but in an imbalance, with some regions having a low number of publications. The United States was the country with the highest number of publications; 5 of the top 10 institutions in terms of publications belonged to the United States and 2 of the top 10 authors were from the United States, followed by China and England. The USA also had the highest number of citations. The number of citations reflects the influence of the paper in the field and provides an important reference value for further scientific research. The number of citations in a paper reflects the basis and rationale of the research and provides complete evidence for the academic achievements. From different perspectives, the United States has great concern and investigation in the field of prediabetes, is recognized by other researchers, and has a great influence on this research field. By contrast, the discrepancy between the number and H index in China suggested that we should further improve the quality of research achievements in the subject area of prediabetes. The same situation was observed in Japan.

We also found close cooperation between countries, especially between the United States and China, which emphasize cooperation with other countries and between China and the United States. This finding indicated that good national cooperation and communication help the development of the subject area. Except for China and India, the top 10 countries in terms of the number of publications belong to developed countries, indicating that China and India are at the front of developing countries in the field of prediabetes research.

In terms of institutions and affiliations, half of the institutions are from the US. This finding further illustrated the influence of the US in the subject area. The top institution in terms of the number of publications is the University of Copenhagen from Denmark, followed by Harvard Medical School from the US and Shanghai Jiao Tong University from China. However, Harvard Medical School has the highest H index.

Using Lotka theorem as basis, we identified 1154 core authors with more than 7 publications. This number comprised more than 90% of the total authors, indicating that the field has formed a relatively stable group of author collaborations. The author with the highest number of publications is Tuomilehto, Jaakko, who participated in the ESC Guidelines on diabetes, prediabetes, and cardiovascular diseases developed in collaboration with the EASD.^[16]^ He was also involved in the publication of a clinical cross-sectional study on the prevalence and risk factors of diabetes and prediabetes in Turkish adults.^[17]^ The results of this study provided basis for a national plan and major public health challenge for the prevention and management of diabetes in Turkey. In his recent article, he published a method for screening for glucose abnormalities in patients with coronary heart disease.^[18]^ Rathmann Wolfgang and Peters Annette were the next in the ranking. We also found that 2-thirds of the top ten core authors are from Germany, which may explain why Germany has a high H index and a high quality of published articles even though the number of its publications is comparatively low.

### 4.2. Research focus on prediabetes

The top 10 journals with the highest number of publications all had high IF, and 7 of them had IF scores >5. The top journal was *PLUS ONE*, a comprehensive journal that includes original research in many fields and has a high publication count, mainly in the US and China. The next journals with the most research published by American authors were *DIABETES CARE* and *DIABETES RESEARCH AND CLINICAL PRACTICE. DIABETES CARE*. The most cited articles were written by ADA (title: Standards of Medical Care in Diabetes-2013) and Robles article (title: Classification and Diagnosis of Diabetes), which provided guiding recommendations for the treatment and management of diabetes to reduce inappropriate clinical decision-making practices, decreases treatment costs, and improve the quality and safety of healthcare delivery^[19,20]^ These journals were specialized with high visibility and influence. Academics are likely to proclaim their ideals or opinions in the field of prediabetes to improve their academic standards and scientific competence. On the basis of this trend, the journals shown in Table 5 may be the main journals for future research in this discipline and must be read carefully by researchers working in this field.

The top 10 most frequently cited articles are mainly about the prevalence, trends, risk factors, and prevention of diabetes and prediabetes, indicating that diabetes has become an important issue in global public health. The prediction of prevalence, trends, and risk factors for the disease could help reduce or delay the development of diabetes and improve the quality of life of patients. Xu article has the highest local citation. The study investigated the prevalence and glycemic control of diabetes and prediabetes in Chinese adults through a cross-sectional survey and estimated the prevalence of diabetes and prediabetes to be 11.6% and 50.1%, respectively, indicating that diabetes is a critical public health concern in China and should be taken seriously.^[21]^ Next is the article from Menke (LC: 201), which estimated the prevalence of diabetes in the USA population at 12% to 14% in 2011 to 2012 using the National Health and Nutrition Examination Survey database.^[22]^ The article from Lancet by Zhou focused on predicting prevalence trends from global diabetes research data using Bayesian hierarchical models and found that the prevalence of diabetes has increased significantly since 1980, particularly in women, and showed geographic differences.^[23]^ This finding indicated that researchers are concerned about the prevalence, treatment guidelines, and definitions of diabetes and prediabetes research.

We found more concern in recent years to study the relationship between prediabetes and diseases such as heart failure and renal dysfunction from the timeline map of co-cited references. Studies demonstrated an association between prediabetes and heart failure,^[24,25]^ and a meta-analysis found that prediabetes was strongly related to a poorer prognosis in patients with heart failure.^[26]^ Although some researchers have concluded that prediabetes is not an independent risk factor for heart failure or other cardiovascular disease in older adults.^[27,28]^ Another study found that prediabetes was connected to a higher lifetime risk of heart failure in middle-aged white adults and black women,^[29]^ and that IFG and IGT were both associated with a modest increase in the risk of cardiovascular disease.^[30]^ Therefore, prediabetes should be considered not only as a high-risk state for the development of diabetes, but also as a risk factor for the development of cardiovascular disease. In addition, it was found that prediabetes has an independent role in the development of glomerular hyperfiltration and albumin,^[31,32]^ while prediabetes and the risk of renal dysfunction showed a positive correlation.^[33,34]^ Both fasting glucose and HbA1C could be used as predictors of reduced renal function,^[33,35]^ and postprandial glucose had a higher predictive value for renal dysfunction.^[34]^ Moreover, high-density lipoprotein cholesterol (HDL-c) and alanine aminotransferase (ALT) are also strongly associated with prediabetes. A 5-year cohort study in a Chinese adult population revealed that the triglyceride (TG)/HDL-c ratio was positively associated with the incidence of diabetes in prediabetic patients, which was supported by the results of a 10-year follow-up study in Iran.^[36,37]^ However, the aspartate aminotransferase (AST)/ALT ratio was negatively associated with the risk of diabetes in prediabetic patients,^[38,39]^ suggesting that regular monitoring of liver function levels in prediabetic patients could help prevent or slow the progression of diabetes.

From the timeline map of keywords, we found that studies have focused on insulin sensitivity, prediabetic high-risk diseases (polycystic ovary syndrome (PCOS), coronary artery disease, dyslipidemia) and lifestyle interventions. Zhu found differences in prediabetes by different criteria in a longitudinal study and stated that targeted prevention should be categorized by impaired fasting glucose, impaired glucose tolerance, and elevated HbA1c.^[40]^ Patients with prediabetes are at high risk of developing diabetes and have high prevalence of dyslipidemia, high levels of atherogenic lipids, and increased risk of atherosclerotic cardiovascular disease. For patients with elevated IGT and/or 7-hour plasma glucose, the same intensive treatment of dyslipidemia as recommended for diabetes should be considered.^[41]^ Patients with PCOS are at high risk for prediabetes and diabetes, and their adverse cardiovascular risk profile is similar to that of patients with prediabetes and type 2 diabetes.^[42]^ HbA1C should be used to detect abnormal blood glucose in adolescents with PCOS; meanwhile, fasting glucose is not sufficiently sensitive to detect diabetes in the PCOS population, and routine OGTT is not sufficiently diagnostic for normal-weight women.^[43–45]^ Lifestyle interventions focus on lowering blood glucose levels through interventions, such as weight loss, dietary changes, and increased physical activity, with significant reductions in diabetes risk.^[46,47]^ Elizabeth performed early time-restricted feeding in men with prediabetes and found that it increased insulin sensitivity, improved beta-cell function and weight loss, and reduced blood pressure and oxidative stress.^[48]^ Recent research has focused on metabolism-related pathways and phenotypes and gut microbes. The Allin study found that prediabetic patients had an abnormal gut microbiota characterized by a reduced abundance of the genus *Clostridium* and the mucin-degrading bacterium *A. muciniphila*.^[49]^ Zhang study also demonstrated that gut microbial changes can respond to the progression of glucose intolerance, and metabolic parameters (FPG, C-reactive protein) are closely associated with gut microbes.^[50]^ Personalized dietary interventions may alter the population configuration of gut microbes, thereby ameliorating postprandial glucose elevation.^[51]^

### 4.3. Strengths and limitations

This study used bibliometric analysis for the first time to provide insights into the global status and trends in prediabetes research. The findings could help researchers who are interested in prediabetes to establish a clear framework of the existing research in this field and to gain insight into the development process in the last decade. Bibliometric analysis is objective, comprehensive and extensive, contributing to the reliability of the data. However, some limitations should be addressed. First, the publication deadline was March 10, 2023, but the WOS database would also keep updating. Newly published literature is already available online on the journal website, and this section is missing from this manuscript. Second, this study was restricted to journal articles included in the WOS, the dominant database for the bibliometric analysis, omitting a few papers that were not included. Third, we used subject terms to identify articles in the database for this study, limiting the language of the article to “English” and the type of article to “Articles and Review Articles,” which may not include some productions. Despite these limitations, we believe that this study could provide an overview of prediabetes research and general trends over the past decade.

## 5. Conclusion

As an intermediate state of the disease, prediabetes has a broad research scope and worth. Therefore, studies from the perspective of bibliometrics and scientometrics are extremely valuable to sort out and provide direction and ideas for research in this field. The clustering and timeline analysis of high-frequency keywords and co-cited references in this study may help researchers understand that the hotspots of prediabetes research are insulin sensitivity and intervention and prevention of high-risk diseases in prediabetes. Meanwhile, research hotspots in recent years include metabolism-related pathways, phenotypes and biomarkers, and gut microbes. This study analyzed the core authors and core journals in the field of prediabetes, allowing researchers to quickly find the literature they want to refer to when conducting research. The latest research and guideline changes can be found in *DIABETES CARE, JOURNAL OF CLINICAL ENDOCRINOLOGY,* and *DIABETOLOGIA*. Tuomilehto, Jaakko and Rathmann, Wolfgang and Peters, Annette are the core authors in the field. This work also provides a guide for researchers in choosing journals when submitting papers on related topics.

In the next study, we need to integrate the literature from multiple databases to make the screening data as comprehensive as possible, and actively communicate with academics in the field to understand the cutting-edge developments in the field and form a more objective and rational perception of the research field.

## Author contributions

**Conceptualization:** Guoyu Wang, Min Jiang.

**Data curation:** Xinxin Liu, Siyi Ma.

**Formal analysis:** Guoyu Wang, Yafei Chen.

**Resources:** Xinxin Liu, Siyi Ma.

**Software:** Guoyu Wang, Yafei Chen.

**Supervision:** Min Jiang.

**Validation:** Guoyu Wang, Xinxin Liu, Siyi Ma.

**Visualization:** Guoyu Wang, Yafei Chen.

**Writing – original draft:** Guoyu Wang, Yafei Chen.

**Writing – review & editing:** Guoyu Wang, Yafei Chen, Min Jiang.
